# 4-Methyl­anilinium tetra­fluoro­borate 18-crown-6 clathrate

**DOI:** 10.1107/S1600536810019033

**Published:** 2010-05-29

**Authors:** Jia-Zhen Ge, Min-Min Zhao

**Affiliations:** a Ordered Matter Science Research Center, College of Chemistry and Chemical, Engineering, Southeast University, Nanjing 211189, People’s Republic of China

## Abstract

In the title compound, C_7_H_10_N^+^·BF_4_^−^·C_12_H_24_O_6_, the proton­ated 4-methyl­anilinium cation inter­acts with 18-crown-6 forming a rotator–stator structure, (C_6_H_4_CH_3_NH_3_^+^)(18-crown-6), through three bifurcated N—H⋯(O,O) hydrogen bonds between the ammonium groups of the cations (–NH_3_) and the O atoms of the crown ether mol­ecule. The BF_4_^−^ anions, the methyl group and the protonated –NH_3_ groups of the 4-methylanilinium lie on a dual axis of rotation. The 18-crown-6 unit is perpendicular to the dual axis of rotation and the mirror plane which contains the dual axis of rotation. The benzene ring of 4-methylanilinium is perpendicular to the mirror plane and parallel to the dual axis.

## Related literature

For a similar 18-crown-6 clathrate, see: Pedersen *et al.* (1967 ▶). For ferroelectric properties, see: Fu *et al.* (2007 ▶); Ye *et al.* (2009 ▶).; Zhang *et al.* (2009 ▶). 
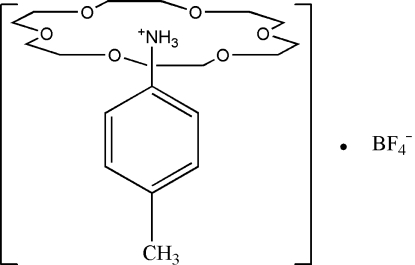


## Experimental

### 

#### Crystal data

C_7_H_10_N^+^·BF_4_^−^·C_12_H_24_O_6_
*M* *_r_* = 459.28
Orthorhombic, 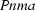

*a* = 15.439 (3) Å
*b* = 11.616 (2) Å
*c* = 13.071 (3) Å
*V* = 2344.2 (8) Å^3^
*Z* = 4
Mo *K*α radiation
μ = 0.11 mm^−1^
*T* = 293 K
0.20 × 0.20 × 0.20 mm

#### Data collection

Rigaku SCXmini diffractometer
Absorption correction: multi-scan (*CrystalClear*; Rigaku, 2005 ▶) *T*_min_ = 0.977, *T*_max_ = 0.977
23254 measured reflections
2816 independent reflections
1541 reflections with *I* > 2σ(*I*)
*R*_int_ = 0.086

#### Refinement

*R*[*F* ^2^ > 2σ(*F* ^2^)] = 0.067
*wR*(*F* ^2^) = 0.209
*S* = 1.02
2816 reflections
154 parameters
H-atom parameters constrained
Δρ_max_ = 0.30 e Å^−3^
Δρ_min_ = −0.23 e Å^−3^

### 

Data collection: *CrystalClear* (Rigaku, 2005 ▶); cell refinement: *CrystalClear*; data reduction: *CrystalClear*; program(s) used to solve structure: *SHELXS97* (Sheldrick, 2008 ▶); program(s) used to refine structure: *SHELXL97* (Sheldrick, 2008 ▶); molecular graphics: *SHELXTL* (Sheldrick, 2008 ▶); software used to prepare material for publication: *PRPKAPPA* (Ferguson, 1999 ▶).

## Supplementary Material

Crystal structure: contains datablocks I, global. DOI: 10.1107/S1600536810019033/jh2160sup1.cif

Structure factors: contains datablocks I. DOI: 10.1107/S1600536810019033/jh2160Isup2.hkl

Additional supplementary materials:  crystallographic information; 3D view; checkCIF report

## Figures and Tables

**Table 1:** Hydrogen-bond geometry (Å, °)

*D*—H⋯*A*	*D*—H	H⋯*A*	*D*⋯*A*	*D*—H⋯*A*
N1—H1*C*⋯O2^i^	0.89	2.21	2.958 (2)	141
N1—H1*C*⋯O3^i^	0.89	2.22	2.960 (2)	140
N1—H1*D*⋯O4	0.89	2.16	2.887 (4)	138
N1—H1*D*⋯O3	0.89	2.22	2.960 (2)	141
N1—H1*E*⋯O1	0.89	2.18	2.920 (4)	140
N1—H1*E*⋯O2	0.89	2.22	2.958 (2)	140

Symmetry code: (i) 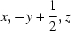.

**Fig. 1.**
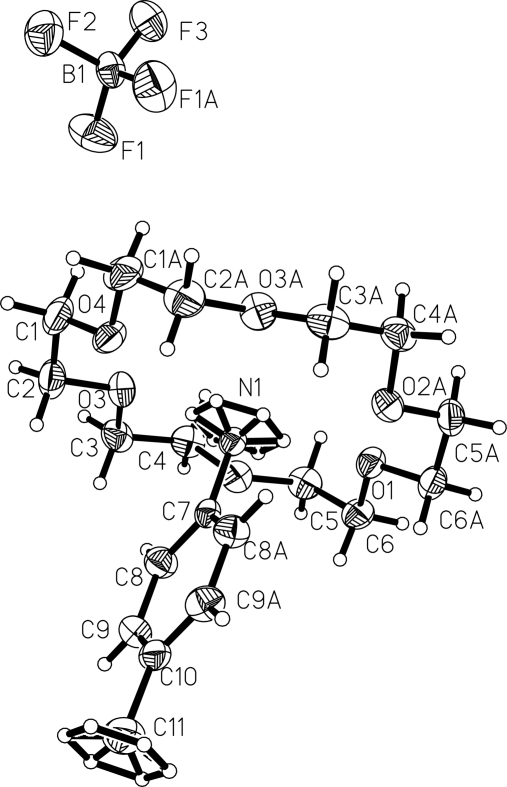
The molecular structure of the title compound, with the atomic numbering scheme. Displacement ellipsoids are drawn at the 30% probability level.

**Fig. 2.**
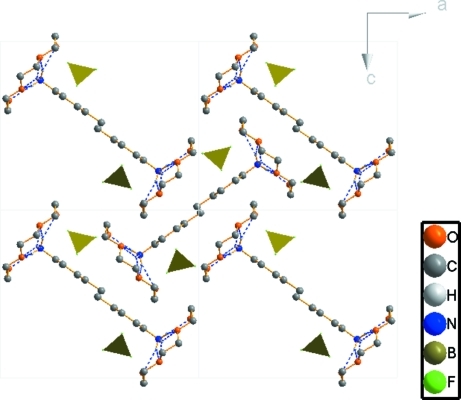
A view of the packing of the title compound, stacking along the b axis. Dashed lines indicate hydrogen bonds.

## supplementary crystallographic information

### Comment

The crown ethers were of a great interest since their discovery had been reported by Pedersen (Pedersen *et al.* 1967). The ability of these macrocycles to form non-covalent,H-bonding complexes with ammonium cations has been actively investigated. Both the size of the crown ether and the nature of the ammonium cation (-NH_4_^+^, RNH_3_^+^, *etc*) can influence on the stoichiometry and stability of these host-guest complexes. The host molecules combine with the guest species by intermolecular interaction, and if the host molecule possess some specific sites, it is easy to realise high selectivity in ion or molecular recognitions.18-crown-6 have the highest affinity for ammonium cation RNH_3_^+^, while most studies of 18-crown-6 and its derivatives invariably showed a 1:1 stoichiometry with RNH_3_^+^ cations.

The title compound dielectric permittivity is tested to systematically investigate the ferroelectric phase transitions materials (Fu *et al.* 2007; Ye *et al.* 2009; Zhang *et al.* 2009). The title compound have no dielectric anomalies with the value of 4-5 and 6-8 under 1M Hz in the temperature from 80 to 300 K and 300 to 473 K (the compound m.p.> 473 K), respectively, suggesting that the compound should be no distinct phase transition occurred within the measured temperature range.

The the title compound is composed of cationic [(C_6_H_4_CH_3_NH_3_) (18-Crown-6)]^+^ and one isolated anionic [BF_4_]^-^ (Fig 1). The protonated 4-methylaniline [C_6_H_4_CH_3_NH_3_]^+^ and 18-crown-6 form superamolecular rotator-stator structure by forming hydrogen-bond (N—H···O) between the ammonium moieties of (-NH_3_^+^) cations and each of the six oxygen atoms of crown ethers. The intramolecular N—H···O hydrogen bonding length are within the usual range: 2.887 (4) and 2.960 (2) Å. The crown ring show slight distortion. The six oxygen atoms of the crown ether lie approximately in a plane. The C—N bonds of [C_6_H_4_CH_3_NH_3_]^+^ were almost perpendicular to the mean oxygen plane.

The typical B—F bond lengths in the tetrahedral coordinate anion [BF_4_]^-^ are within 1.374 (4)-1.393 (5) Å. The F—B—F bond angles indicate little distortion from a regular tetrahedron [spread of values 107.3 (4)-111.6 (4)°].

Fig. 2 shows a view down the *b* axis. The couples of head-to-head rotator-stator cations almost paralleling and plumbing the (1 0 1) direction are alternating arranged. The anions [BF_4_]^-^ inhabit the cave formed by the couples of head-to-head rotator-stator cations. The title compound was stabilized by intramolecular N—H···O hydrogen bonds, but no intermolecular hydrogen bond was observed.

### Experimental

4-methylaniline(2 mmol, 0.214 g) and excessive tetrafluoroborate (4 mmol, 0.348 g) were dissolved in methanol solution. Then 18-crown-6 (2 mmol, 0.528 g) was added to the mixture. The precipitate was filtered and washed with a small amount of methanol. Single crystals suitable for X-ray diffraction analysis were obtained from slow evaporation of methanol solution at room temperature after two days.

### Refinement

All the C—H hydrogen atoms were calculated geometrically and with C—H distances ranging from 0.93 to 0.97 Å and were allowed to ride on the C and O atoms to which they are bonded. With which *U*_iso_(H) = 1.2Ueq(C).

### Crystal data

**Table:**

C_7_H_10_N^+^·BF_4_^−^·C_12_H_24_O_6_	*F*(000) = 976
*M**_r_* = 459.28	*D*_x_ = 1.301 Mg m^−^^3^
Orthorhombic, *P**n**m**a*	Mo *K*α radiation, λ = 0.71073 Å
Hall symbol: -P 2ac 2n	Cell parameters from 2816 reflections
*a* = 15.439 (3) Å	θ = 3.1–27.5°
*b* = 11.616 (2) Å	µ = 0.11 mm^−^^1^
*c* = 13.071 (3) Å	*T* = 293 K
*V* = 2344.2 (8) Å^3^	Block, pale yellow
*Z* = 4	0.20 × 0.20 × 0.20 mm

### Data collection

**Table:**

Rigaku SCXmini diffractometer	2816 independent reflections
Radiation source: fine-focus sealed tube	1541 reflections with *I* > 2σ(*I*)
graphite	*R*_int_ = 0.086
Detector resolution: 13.6612 pixels mm^-1^	θ_max_ = 27.5°, θ_min_ = 3.1°
ω scans	*h* = −20→20
Absorption correction: multi-scan (*CrystalClear*; Rigaku, 2005)	*k* = −15→15
*T*_min_ = 0.977, *T*_max_ = 0.977	*l* = −16→16
23254 measured reflections	

### Refinement

**Table:**

Refinement on *F*^2^	Primary atom site location: structure-invariant direct methods
Least-squares matrix: full	Secondary atom site location: difference Fourier map
*R*[*F*^2^ > 2σ(*F*^2^)] = 0.067	Hydrogen site location: inferred from neighbouring sites
*wR*(*F*^2^) = 0.209	H-atom parameters constrained
*S* = 1.02	*w* = 1/[σ^2^(*F*_o_^2^) + (0.0947*P*)^2^ + 0.766*P*] where *P* = (*F*_o_^2^ + 2*F*_c_^2^)/3
2816 reflections	(Δ/σ)_max_ < 0.001
154 parameters	Δρ_max_ = 0.30 e Å^−^^3^
0 restraints	Δρ_min_ = −0.23 e Å^−^^3^

### Special details

**Table:**

Geometry. All esds (except the esd in the dihedral angle between two l.s. planes) are estimated using the full covariance matrix. The cell esds are taken into account individually in the estimation of esds in distances, angles and torsion angles; correlations between esds in cell parameters are only used when they are defined by crystal symmetry. An approximate (isotropic) treatment of cell esds is used for estimating esds involving l.s. planes.
Refinement. Refinement of *F*^2^ against ALL reflections. The weighted *R*-factor wR and goodness of fit *S* are based on *F*^2^, conventional *R*-factors *R* are based on *F*, with *F* set to zero for negative *F*^2^. The threshold expression of *F*^2^ > σ(*F*^2^) is used only for calculating *R*-factors(gt) etc. and is not relevant to the choice of reflections for refinement. *R*-factors based on *F*^2^ are statistically about twice as large as those based on *F*, and *R*- factors based on ALL data will be even larger.

### Fractional atomic coordinates and isotropic or equivalent isotropic displacement parameters (Å^2^)

**Table:**

	*x*	*y*	*z*	*U*_iso_*/*U*_eq_	Occ. (<1)
O1	0.45735 (17)	0.2500	0.85026 (19)	0.0531 (7)	
O2	0.39198 (12)	0.03619 (16)	0.78258 (14)	0.0548 (5)	
O3	0.30174 (13)	0.04501 (16)	0.59530 (15)	0.0607 (6)	
O4	0.21893 (18)	0.2500	0.5296 (2)	0.0630 (8)	
C5	0.4704 (2)	0.0478 (3)	0.8399 (2)	0.0615 (8)	
H5A	0.5189	0.0592	0.7937	0.074*	
H5B	0.4809	−0.0217	0.8792	0.074*	
C3	0.3162 (2)	−0.0608 (2)	0.6496 (2)	0.0640 (8)	
H3A	0.3188	−0.1245	0.6017	0.077*	
H3B	0.2689	−0.0747	0.6968	0.077*	
C4	0.3988 (2)	−0.0519 (3)	0.7065 (2)	0.0617 (8)	
H4A	0.4118	−0.1250	0.7390	0.074*	
H4B	0.4455	−0.0337	0.6596	0.074*	
C2	0.2237 (2)	0.0450 (3)	0.5374 (3)	0.0719 (9)	
H2A	0.1742	0.0476	0.5831	0.086*	
H2B	0.2199	−0.0249	0.4971	0.086*	
C6	0.4625 (2)	0.1481 (2)	0.9098 (2)	0.0587 (8)	
H6A	0.4109	0.1403	0.9516	0.070*	
H6B	0.5124	0.1517	0.9548	0.070*	
C1	0.2230 (2)	0.1471 (3)	0.4689 (2)	0.0770 (10)	
H1A	0.2750	0.1478	0.4273	0.092*	
H1B	0.1732	0.1435	0.4236	0.092*	
N1	0.29810 (18)	0.2500	0.7297 (2)	0.0432 (7)	
H1C	0.3138	0.3222	0.7167	0.065*	0.50
H1D	0.2860	0.2142	0.6712	0.065*	0.50
H1E	0.3412	0.2136	0.7613	0.065*	0.50
C7	0.2212 (2)	0.2500	0.7954 (2)	0.0406 (8)	
C8	0.18519 (19)	0.1469 (3)	0.8255 (2)	0.0566 (7)	
H8A	0.2099	0.0775	0.8054	0.068*	
C10	0.0727 (2)	0.2500	0.9169 (3)	0.0562 (11)	
C9	0.11146 (19)	0.1480 (3)	0.8863 (2)	0.0624 (8)	
H9A	0.0874	0.0784	0.9071	0.075*	
C11	−0.0088 (3)	0.2500	0.9817 (4)	0.0863 (15)	
H11A	−0.0260	0.3279	0.9954	0.129*	0.50
H11B	0.0023	0.2110	1.0450	0.129*	0.50
H11C	−0.0544	0.2111	0.9455	0.129*	0.50
B1	0.3985 (3)	0.2500	0.2064 (4)	0.0572 (12)	
F3	0.48142 (17)	0.2500	0.1644 (2)	0.0831 (8)	
F1	0.38646 (15)	0.1522 (2)	0.2638 (2)	0.1101 (8)	
F2	0.3400 (2)	0.2500	0.1254 (2)	0.0999 (10)	

### Atomic displacement parameters (Å^2^)

**Table:**

	*U*^11^	*U*^22^	*U*^33^	*U*^12^	*U*^13^	*U*^23^
O1	0.0597 (17)	0.0518 (15)	0.0479 (15)	0.000	−0.0094 (12)	0.000
O2	0.0579 (12)	0.0441 (10)	0.0624 (12)	0.0055 (8)	0.0011 (9)	−0.0021 (9)
O3	0.0587 (12)	0.0543 (12)	0.0692 (13)	−0.0104 (9)	−0.0082 (10)	−0.0084 (10)
O4	0.0669 (19)	0.080 (2)	0.0418 (15)	0.000	−0.0083 (13)	0.000
C5	0.0575 (18)	0.0592 (18)	0.0677 (19)	0.0124 (14)	−0.0051 (15)	0.0141 (15)
C3	0.074 (2)	0.0441 (16)	0.074 (2)	−0.0127 (14)	0.0129 (16)	−0.0112 (14)
C4	0.071 (2)	0.0456 (16)	0.0688 (19)	0.0061 (14)	0.0090 (16)	−0.0020 (14)
C2	0.061 (2)	0.080 (2)	0.074 (2)	−0.0116 (16)	−0.0106 (17)	−0.0257 (19)
C6	0.0621 (18)	0.0637 (19)	0.0503 (15)	0.0038 (14)	−0.0086 (13)	0.0117 (15)
C1	0.070 (2)	0.109 (3)	0.0524 (17)	−0.0058 (19)	−0.0146 (15)	−0.020 (2)
N1	0.0446 (17)	0.0441 (16)	0.0411 (16)	0.000	−0.0027 (13)	0.000
C7	0.0379 (19)	0.047 (2)	0.0374 (18)	0.000	−0.0074 (15)	0.000
C8	0.0599 (18)	0.0526 (17)	0.0574 (17)	−0.0035 (13)	0.0064 (14)	−0.0007 (13)
C10	0.040 (2)	0.086 (3)	0.042 (2)	0.000	−0.0028 (17)	0.000
C9	0.0597 (19)	0.068 (2)	0.0599 (17)	−0.0157 (15)	0.0048 (14)	0.0030 (15)
C11	0.067 (3)	0.122 (4)	0.070 (3)	0.000	0.009 (3)	0.000
B1	0.049 (3)	0.047 (3)	0.076 (3)	0.000	−0.010 (2)	0.000
F3	0.0672 (17)	0.0736 (18)	0.109 (2)	0.000	0.0040 (15)	0.000
F1	0.1019 (17)	0.0984 (17)	0.1301 (18)	−0.0018 (13)	0.0172 (14)	0.0433 (15)
F2	0.081 (2)	0.114 (2)	0.105 (2)	0.000	−0.0178 (17)	0.000

### Geometric parameters (Å, °)

**Table:**

O1—C6^i^	1.419 (3)	C1—H1A	0.9700
O1—C6	1.419 (3)	C1—H1B	0.9700
O2—C5	1.431 (3)	N1—C7	1.465 (4)
O2—C4	1.431 (3)	N1—H1C	0.8900
O3—C2	1.423 (3)	N1—H1D	0.8900
O3—C3	1.436 (4)	N1—H1E	0.8900
O4—C1	1.436 (4)	C7—C8	1.378 (3)
O4—C1^i^	1.436 (4)	C7—C8^i^	1.378 (3)
C5—C6	1.486 (4)	C8—C9	1.388 (4)
C5—H5A	0.9700	C8—H8A	0.9300
C5—H5B	0.9700	C10—C9^i^	1.386 (4)
C3—C4	1.480 (4)	C10—C9	1.386 (4)
C3—H3A	0.9700	C10—C11	1.517 (6)
C3—H3B	0.9700	C9—H9A	0.9300
C4—H4A	0.9700	C11—H11A	0.9600
C4—H4B	0.9700	C11—H11B	0.9600
C2—C1	1.487 (5)	C11—H11C	0.9600
C2—H2A	0.9700	B1—F1	1.374 (4)
C2—H2B	0.9700	B1—F1^i^	1.374 (4)
C6—H6A	0.9700	B1—F3	1.392 (5)
C6—H6B	0.9700	B1—F2	1.393 (5)
			
C6^i^—O1—C6	113.0 (3)	O4—C1—H1A	109.8
C5—O2—C4	111.7 (2)	C2—C1—H1A	109.8
C2—O3—C3	113.2 (2)	O4—C1—H1B	109.8
C1—O4—C1^i^	112.7 (3)	C2—C1—H1B	109.8
O2—C5—C6	109.0 (2)	H1A—C1—H1B	108.2
O2—C5—H5A	109.9	C7—N1—H1C	109.5
C6—C5—H5A	109.9	C7—N1—H1D	109.5
O2—C5—H5B	109.9	H1C—N1—H1D	109.5
C6—C5—H5B	109.9	C7—N1—H1E	109.5
H5A—C5—H5B	108.3	H1C—N1—H1E	109.5
O3—C3—C4	108.8 (2)	H1D—N1—H1E	109.5
O3—C3—H3A	109.9	C8—C7—C8^i^	120.7 (3)
C4—C3—H3A	109.9	C8—C7—N1	119.65 (17)
O3—C3—H3B	109.9	C8^i^—C7—N1	119.65 (17)
C4—C3—H3B	109.9	C7—C8—C9	119.1 (3)
H3A—C3—H3B	108.3	C7—C8—H8A	120.4
O2—C4—C3	109.6 (2)	C9—C8—H8A	120.4
O2—C4—H4A	109.7	C9^i^—C10—C9	117.4 (4)
C3—C4—H4A	109.7	C9^i^—C10—C11	121.28 (18)
O2—C4—H4B	109.7	C9—C10—C11	121.28 (18)
C3—C4—H4B	109.7	C10—C9—C8	121.8 (3)
H4A—C4—H4B	108.2	C10—C9—H9A	119.1
O3—C2—C1	109.0 (3)	C8—C9—H9A	119.1
O3—C2—H2A	109.9	C10—C11—H11A	109.5
C1—C2—H2A	109.9	C10—C11—H11B	109.5
O3—C2—H2B	109.9	H11A—C11—H11B	109.5
C1—C2—H2B	109.9	C10—C11—H11C	109.5
H2A—C2—H2B	108.3	H11A—C11—H11C	109.5
O1—C6—C5	108.7 (2)	H11B—C11—H11C	109.5
O1—C6—H6A	109.9	F1—B1—F1^i^	111.6 (4)
C5—C6—H6A	109.9	F1—B1—F3	109.9 (2)
O1—C6—H6B	109.9	F1^i^—B1—F3	109.9 (2)
C5—C6—H6B	109.9	F1—B1—F2	109.1 (3)
H6A—C6—H6B	108.3	F1^i^—B1—F2	109.1 (3)
O4—C1—C2	109.4 (2)	F3—B1—F2	107.3 (4)

Symmetry codes: (i) *x*, −*y*+1/2, *z*.

### Hydrogen-bond geometry (Å, °)

**Table:**

*D*—H···*A*	*D*—H	H···*A*	*D*···*A*	*D*—H···*A*
N1—H1C···O2^i^	0.89	2.21	2.958 (2)	141
N1—H1C···O3^i^	0.89	2.22	2.960 (2)	140
N1—H1D···O4	0.89	2.16	2.887 (4)	138
N1—H1D···O3	0.89	2.22	2.960 (2)	141
N1—H1E···O1	0.89	2.18	2.920 (4)	140
N1—H1E···O2	0.89	2.22	2.958 (2)	140

Symmetry codes: (i) *x*, −*y*+1/2, *z*.
